# The use of essential oils in chitosan or cellulose‐based materials for the production of active food packaging solutions: a review

**DOI:** 10.1002/jsfa.11918

**Published:** 2022-04-26

**Authors:** Sara Casalini, Marco Giacinti Baschetti

**Affiliations:** ^1^ Department of Civil, Chemical, Environmental and Materials Engineering‐DICAM University of Bologna Bologna Italy

**Keywords:** active packaging, essential oils, antimicrobial activity, biodegradable polymers, nanocellulose, chitosan, shelf‐life

## Abstract

In recent decades, interest in sustainable food packaging systems with additional functionality, able to increase the shelf life of products, has grown steadily. Following this trend, the present review analyzes the state of the art of this active renewable packaging. The focus is on antimicrobial systems containing nanocellulose and chitosan, as support for the incorporation of essential oils. These are the most sustainable and readily available options to produce completely natural active packaging materials. After a brief overview of the different active packaging technologies, the main features of nanocellulose, chitosan, and of the different essential oils used in the field of active packaging are introduced and described. The latest findings about the nanocellulose‐ and chitosan‐based active packaging are then presented. The antimicrobial effectiveness of the different solutions is discussed, focusing on their effect on other material properties. The effect of the different inclusion strategies is also reviewed considering both *in vivo* and *in vitro* studies, in an attempt to understand more promising solutions and possible pathways for further development. In general, essential oils are very successful in exerting antimicrobial effects against the most diffused gram‐positive and gram‐negative bacteria, and affecting other material properties (tensile strength, water vapor transmission rate) positively. Due to the wide variety of biopolymer matrices and essential oils available, it is difficult to create general guidelines for the development of active packaging systems. However, more attention should be dedicated to sensory analysis, release kinetics, and synergetic action of different essential oils to optimize the active packaging on different food products. © 2022 The Authors. *Journal of The Science of Food and Agriculture* published by John Wiley & Sons Ltd on behalf of Society of Chemical Industry.

## INTRODUCTION

In recent years, rapid social development has led to a substantial increase in the consumption of all types of goods^1^ and huge amounts of products are shipped around the world every day to meet the demands of consumers.^2^ The food market is one of the most important and globalized markets, as it is now possible to find in every city foods coming literally from any part of the globe.^3^

The expansion of the food market has brought a parallel increase in the importance of food packaging, both in terms of quantity and efficiency.^4^ New types of packaging have been developed following the need to increase the shelf life of the products, to ensure no damage during transport and storage,^5^ and to reduce the environmental impact through the use of renewable or sustainable materials.^6^ The global food packaging market (growing from 293 to 423 billion dollars in the period 2018–2020, according to Zion Market Research^7^) covers 42% of the global polymer market. It reached production rates of up to 100 million tonnes year^−1^ ^8^ and caused many well known environmental problems.

For all these reasons, in recent years, research on more effective packaging has gained much more importance as a result of attempts to obtain better performance with greener materials.^9^ Among the different approaches, particular interest has been devoted to the development of smart and active systems,^10^, ^11^ especially when coupled with their inclusion in environmentally friendly packaging solutions.^12^

**Figure 1:**
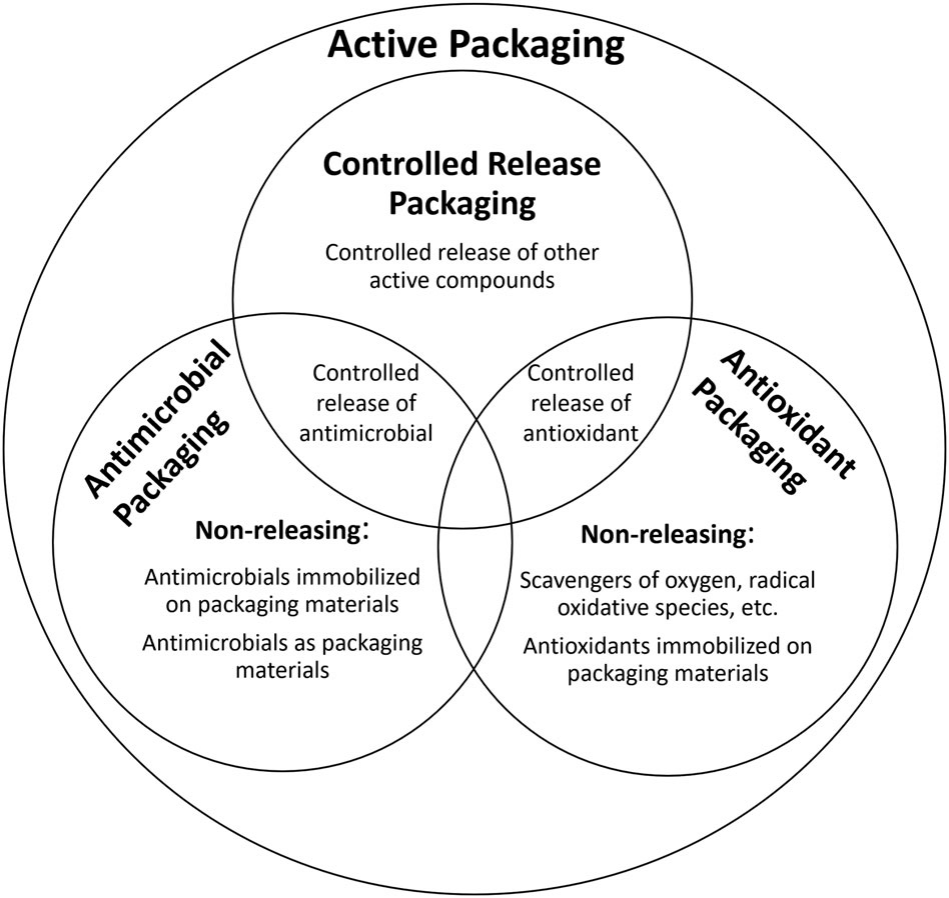
Active packaging. These systems can be applied through different techniques such as controlled release packaging, antimicrobial packaging, and antioxidant packaging.

Figure 1 shows a possible classification of the different types of active packaging technologies.^13^, ^14^ They can be divided in active scavenging systems, active release systems, and non‐releasing systems. The first group includes modified atmosphere packaging (MAP) and other packaging involving the use of absorbents. For example, oxygen or moisture scavengers^15^, ^16^ aim to reduce bacterial growth by maintaining an adverse environment for their development within the package. The second type acts, instead, through the release of molecules,^17^ which prevent food spoilage.^10^ Finally, much of the existing antimicrobial packaging belongs to the third type and is based on metal nanoparticles (silver, gold, and zinc), metal oxide nanomaterials (silicon and magnesium, titanium, zinc, etc.),^18^ and carbon nanotubes, which are very effective in developing antimicrobial effects once immobilized on the package surface.^8^, ^14^, ^19^

Among the different possible solutions, there has recently been an increasing interest in active release systems based on the use of essential oils (EOs) as active compounds. These are secondary metabolites that can be obtained from different parts of scented plants.^20^ They provide antioxidant and/or antimicrobial effects^21^, ^22^ and can interact with the packaging material, reducing water vapor permeability and influencing mechanical and optical properties.^23^ These capabilities, as well as the fact that they are natural and intrinsically sustainable, made them the first choice for many active packaging solutions tested in recent years.

**Figure 2:**
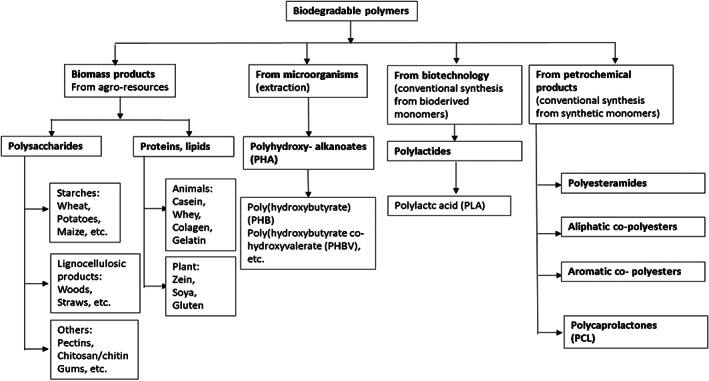
Classification of biodegradable polymers.^26^ Biodegradable polymers can be obtained from different sources such as biomass, microorganisms, and petrochemical products.

Indeed, it is important to recall that, with the expansion of the food packaging market, not only were food‐quality issues addressed but environmental concerns regarding the materials used were also recognized. The use of sustainable solutions has been a priority, both in terms of active substances, such as EOs, and of packaging matrix, through the use of renewable and biodegradable materials such as, for example, biopolymers.^24^, ^25^ The wide range of these promising bio‐derived materials can be categorized by their method of production, as shown in Fig. 2.^26^ If they are directly extracted from biomass, they can be divided into polysaccharide films (such as starch, cellulose and derivatives, chitosan and alginate), protein films (soy protein and wheat gluten), lipid films (fatty acids and resins), and composite films.^9^ Otherwise, they can be synthesized from bio‐derived monomers, such as poly‐lactic acid (PLA), or produced directly from microorganisms (like polyhydroxyalkanoates).^24^ Among these materials, particular attention has been attracted by nanocellulose^27^, ^28^, ^29^ and chitosan^30^, ^31^ due to their high versatility and abundance in nature. These two materials, used alone or in conjunction with other biopolymers, have become the basis of several biomaterials for packaging applications.^24^

The present review summarizes some of the most interesting current trends in active packaging research. The focus is on the latest outcomes concerning nanocellulose and chitosan‐based active packaging and the use of different essential oils as antimicrobial agents.

The chitosan and nanocellulose structures and main properties are presented to provide an understanding of their importance as fillers or substrates. Other biopolymers, such as PLA and alginate, will also be considered as they are often coupled with the previous ones in packaging materials. The latest studies on nanocellulose‐based and chitosan‐based active packaging systems will be also discussed, with a focus on the essential oils’ effects on the mechanical and barrier properties of the final material. Antimicrobial effects against the most common food spoilage bacteria and the direct application to some food products will be presented.

## ACTIVE PACKAGING BIOPOLYMERS

In recent years a wide range of biomaterials have been considered for packaging application to reduce the use of oil‐based plastics and to increase the sustainability of the packages.^32^ Among these biomaterials, cellulose and chitosan seem the most promising biopolymers because they are biodegradable and biobased. Moreover, they are among the most abundant polymers present in nature and can be obtained from wastes, strengthening the idea of the circular economy.

### Cellulose

**Figure 3:**
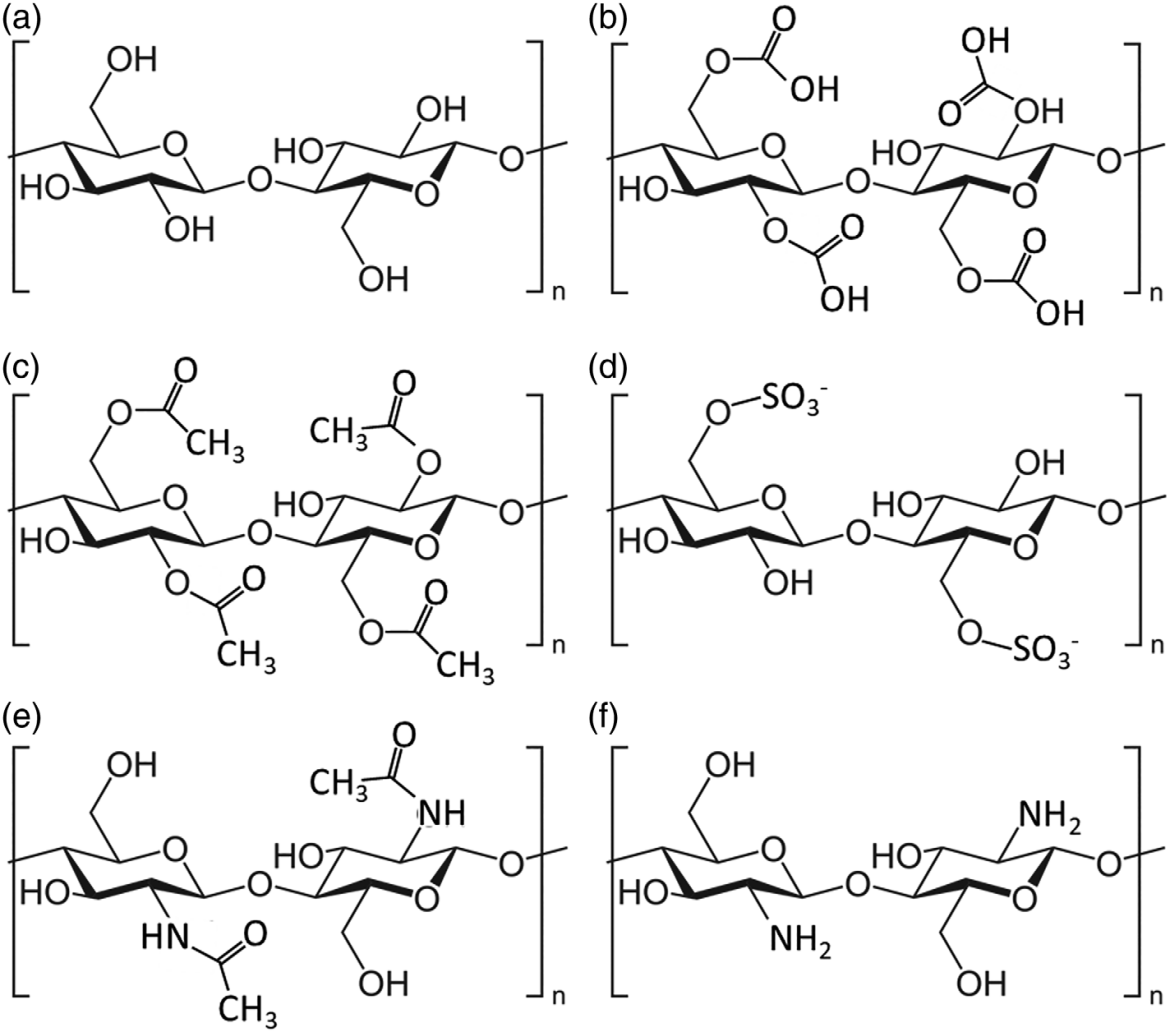
Structure of cellulose materials and chitosan (a) cellulose, (b) carboxymethyl cellulose (CMC), (c) cellulose acetate (CA), (d) cellulose sulfate (CS), (e) chitin, (f) chitosan.

Cellulose is the most abundant and renewable biopolymer in the biosphere. Its size of its global market was USD 346 million in 2021 and it is expected to reach 963 million by 2026.^33^ It is widely distributed in vegetable organisms such as vascular plants, where it has a structural role for the cell walls. Cellulose is a linear homopolysaccharide composed by repeating glucose units (see Fig. 3(a)) and it has been obtained for many years mainly from plant materials. The structure and properties of native cellulose are determined by the isolation process used, which affects the number of inter‐ and intra‐molecular hydrogen bonds, the chain length, the chain length distribution, the crystallinity, and the distribution of functional groups within the repeating units and along the polymer chains.^34^ The hydrogen bonding patterns inside and among cellulose fibers, in particular, are considered to be the main factor that determines its physical and chemical properties.^35^

Cellulose has traditionally been extracted from plants and used to obtain veterinary foods, wood and paper, fibers and clothes, cosmetic and pharmaceutical products.^36^ In recent years, with a view to exploiting its green potential, cellulose has also been obtained from wastes, through different pretreatments of the raw material. For that reason its composition and physical properties could vary influencing the further steps of the process.^27^

Apart from its direct use, cellulose can be modified in many ways obtaining different polymeric materials, such as esters or ethers. These have found wide use in different sectors,^36^, ^37^, ^38^ including active packaging.^39^ Some of the more common cellulose derivatives are shown in Fig. 3(b)–(d).

**Figure 4:**
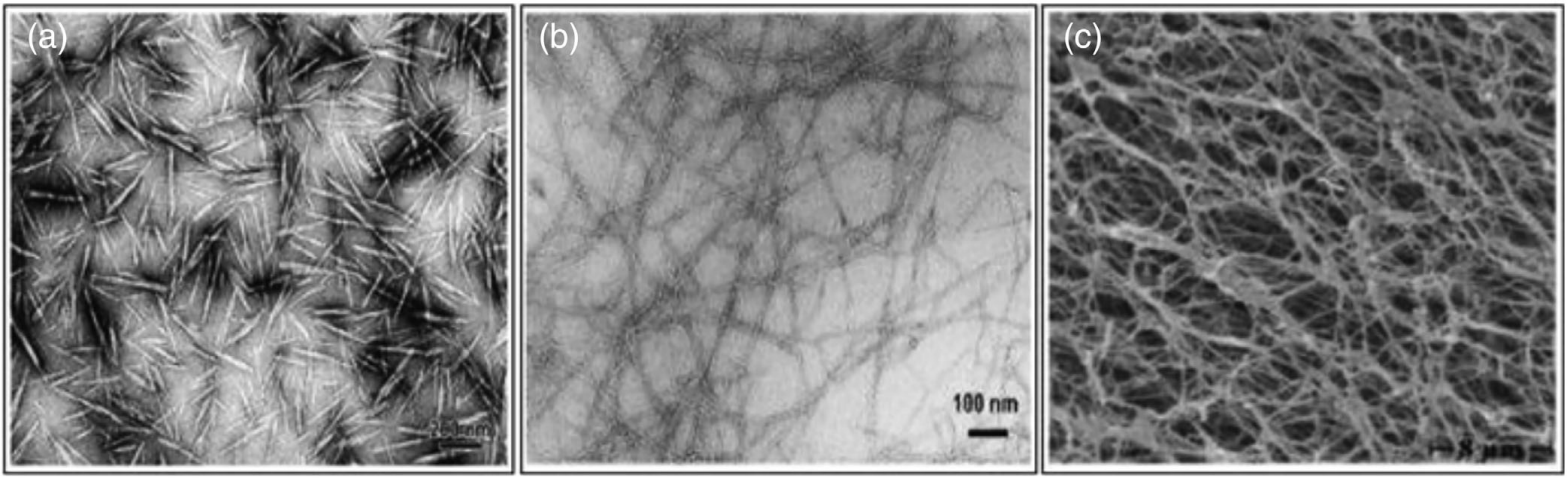
Microscopic image of (a) cellulose nanocrystals, (b) cellulose nanofibrils, and (c) bacterial nanocellulose.^47^

Accordingly with the increasing interest in nanomaterials, cellulose has also been produced with nanoscale dimensions.^40^ Different types of nanocellulose that have generated great interest in a wide range of applications include cellulose nano crystals (CNC), cellulose nano fibrils (CNF), and bacterial nanocellulose (BNC).^41^, ^42^ Briefly, CNF are usually obtained through mechanical homogenization of pristine cellulose fibers, which previously were treated chemically or enzymatically.^43^ Cellulose nano crystals, on the other hand, can be obtained from cellulose by strong acidic treatment, which is able to hydrolyze the amorphous part of the fibers, leaving only the crystalline part of the original fibers.^44^ Finally, BNC comes directly from bacterial metabolism, so it is free from hemicellulose and lignin. This reduces the purification costs and the environmental damage derived from the use of chemicals.^29^, ^45^, ^46^ Figure 4 shows a comparison of the different nanocellulose types described above.

Obviously, the different processes can be optimized depending on the characteristics of the source material and on the final chemical structure needed. Like cellulose, nanocellulose can easily be modified, due to the high number of hydroxyl groups present on the fiber surface.^48^ It allows a great variety of materials to be obtained with modified surface properties that are suitable for many different specific functions.^49^

Nanocellulose is a very versatile material that exhibits good mechanical strength, high barrier properties when in dry conditions, and good biodegradability. It is therefore a strong candidate in food packaging to replace the petroleum‐based products with renewable and biodegradable materials.^50^ A vast literature exists on the use of nanocellulose in packaging, both in pure form as film or coating^51^ or as a reinforcing filler in bio‐composite materials.^29^, ^52^ In the latter case, it is used to increase the strength and the modulus of the matrix and to reduce the water vapor permeability and the oxygen permeability,^53^ or as a stabilizing agent for emulsions.^54^ It was also shown to increase the thermal stability and the water resistance of some biopolymers, such as chitosan,^55^ PLA, and thermoplastic starch.^47^, ^56^

### Chitosan

The second most abundant biological material after cellulose is chitin, the precursor of chitosan. This is a linear highly acetylated polymer (Fig. 3(e)), which is known as chitosan (Fig. 3(f)) when the degree of *N*‐acetylation is lower than 50%.^57^, ^58^ Chitin is mainly obtained from crustacean wastes, through acid and alkaline treatments.^55^ Different factors, such as alkali concentration, incubation time, chitin to alkali ratio, temperature and atmosphere play a role in the alkaline *N*‐deacetylation of chitosan, thus affecting the final properties of the polymer that is obtained.

Chitosan and its derivatives became very useful in many fields like cosmetics, pharmaceuticals, food, agriculture, biomedical and material science due to its biological activity. In fact, those materials are biocompatible, non‐antigenic, non‐toxic, intrinsically antimicrobial, and have a good film‐forming ability.^59^, ^60^ Chitosan‐based active films have been widely studied in recent years as they can be used as antimicrobial agents^61^, ^62^, ^63^ and polymer substrates at the same time.^64^, ^65^ The intrinsic antimicrobial activity of chitosan seems to be addressed to three different mechanisms: ionic surface interaction, penetration of the chitosan in the nuclei of the microorganisms, and the creation of an external barrier inhibiting the nutrients’ contribution.^66^ Moreover, it depends on the polymer molecular weight and on the degree of acetylation.^67^, ^68^

Regarding the food packaging applications, chitosan has been classified as ‘generally recognized as safe’ by the US Food and Drug Administration (FDA) in 2001^69^ and several studies analyze different methods for chitosan film production, in relation to specific food packaging systems.^30^, ^70^, ^71^ As far as active packaging is concerned, then, the effects of chitosan as an antimicrobial preservative were limited to food products with low protein and NaCl content. So the incorporation of antimicrobial agents needs to be considered to extend the protection to all kind of food products.^72^, ^73^

### Other biopolymers

Many other biopolymers have been considered, alone or in combination with nanocellulose or chitosan, to obtain biocomposites with enhanced properties through synergetic effects. In the following sections they will be introduced briefly to explain the importance of these materials used in combination with either nanocellulose or chitosan.

#### Poly‐lactic acid

Poly‐lactic acid is surely one of the most frequently studied biopolymers for food packaging applications. It is biobased and biodegradable, as the lactic acid monomer can be produced from completely renewable resources, and it is also biocompatible.^74^ In fact, it received the approval of the FDA for applications with food contact.

Considering its high transparency, the oil and grease resistance and the optimal organoleptic characteristics, PLA has strong potential for food packaging application. In general, the mechanical and barrier properties of PLA are also remarkable,^75^ but still insufficient to match the performance of many oil‐based polymers. For this reason, several studies have focused on the incorporation of fillers, such as nanocellulose and active agents to impart additional functionalities.^76^ For example, PLA films with 550 g kg^−1^ of NFC showed an increase in their tensile strength and tensile modulus of 59% and 47%, respectively, in comparison with pure PLA films.^77^ Applying a cellulose coating on a PLA substrate allowed a reduction in the oxygen transfer rate of the film of about one order of magnitude even in humid environments (up to 60% RH).^51^

#### Alginate

Alginate is another interesting biopolymer due to its low toxicity and biocompatibility.^78^ It is an example of polysaccharide commonly obtained from the cell wall of brown algae and extracted from seaweed for commercial purposes.^79^ In fact, alginate has already been used to create antibacterial^80^ and antifungal films,^81^ and stimulus‐response drug‐releasing materials, but their application for food packaging is limited due to lack of mechanical strength, as is the case with many other biopolymers.^82^ Several reinforcements were studied to improve alginate's mechanical properties, such as cellulose nanofibrils^83^ or inorganic fillers.^84^ The use of nanocellulose was effective not only for tensile strength (TS) but also for the water vapor barrier properties.^82^, ^85^, ^86^

#### Agar

Agar is a linear polysaccharide extracted from red algae of the class *Rhodophyceae*. It has been used to produce biodegradable films due to its good film‐forming ability, high biocompatibility, and moderate water‐resistant properties.^87^, ^88^, ^89^, ^90^ Agar‐based films also still have some limitations due to their low thermal stability and poor mechanical properties. To improve its properties, agar has been studied in combination with fillers such as metallic nanoparticles and nanoclays,^91^ and in the presence of gelatin^92^ or plant extracts.^93^ Bacterial nanocellulose was also used to reinforce agar‐based edible films.^87^

## ANTIMICROBIAL AGENTS ‐ ESSENTIAL OILS

Antimicrobial agents are compounds used to provide safety assurance, to extend shelf life, and to maintain the quality of food. In fact, when incorporated into the packaging, they are able to inhibit spoilage and suppress the pathogens that are responsible for food‐borne diseases, which can contaminate food products.^94^ Antimicrobial agents can be inorganic compounds such as metals^95^ or metal oxide nanoparticles,^96^ which release antimicrobial ions while directly interacting with the microorganisms. However, the most commercialized products available in the market contain antimicrobial agents such as chlorine dioxide, ethanol and sulfur dioxide, which act in the gas phase of the package.^94^

In recent years there has also been a growing interest in natural antimicrobial agents, due to the lower risk perceived by consumers in their use. The natural compounds used in antimicrobial packaging are biologically derived components, like bacteriocins, enzymes, and plant extracts.^97^, ^98^

In particular, essential oils (EOs) are lipidic extracts from plants that have been studied for many years as additives in films and coatings, to replace synthetic preservatives. In fact, they possess antioxidant and antimicrobial properties naturally, due to the presence of bioactive compounds, such as phenols and terpenoids.^99^ The antimicrobial activity of the essential oils is related to the presence of hydroxyl groups that are able to damage the cell membranes of the pathogens. This results in the release of the cell constituents and in the death of the microorganisms.^20^ For this reason, EOs show a broad antimicrobial spectrum against different pathogenic and spoilage microorganisms, including gram‐negative species such as *Escherichia coli*.^100^

Essential oils differ widely in chemical composition, depending not only on the characteristics of the plant of origin, but also on the part of the plant from which they are extracted and from the extraction process itself.^101^ The qualitative and quantitative differences that could be present may further influence and increase the biological effectiveness. In this regard, Table 1 offers an overview of different essential oils used as antimicrobial agents incorporated in filler/matrix systems to create active biomaterials. These will be explained further, considering their antimicrobial effects as well as their other possible influences on the biocomposite mechanical and barrier properties.

**Table 1:** Different uses of antimicrobial agents incorporated in filler/matrix systems. The first column indicates the antimicrobial agent responsible for the activity of the film, and the second one reports the filler and the matrix where the agent is incorporated. The third column refers to the effects of this integration on the mechanical and chemical properties of the composite, while the fourth one shows the antimicrobial properties and the possible use for active packaging systems. Last column is for references

Antimicrobial agent – essential oil	Filler/matrix	Effect on mechanical and chemical properties	Antimicrobial activity and use	Ref.
Oregano EO	CNC (Pickering emulsion)	Good chemical and thermal stability	*S*. *aureus*, *S*. *cerevisiae*, *E*. *coli* and *B. subtilis*	102
Cinnamon EO	CNC/CNF (Pickering emulsion)	Long‐term emulsion stability	*B. subtilis*	103
Ginger EO, citric acid	CNF (edible coating)	Improved taste, odor, texture, and overall acceptability of the samples	Increase of meat shelf‐life	104
Oregano, Thyme, Cinnamon EOs	Cellulosic pads	Acceptable taste and odor (sensory evaluation)	Meat bacterial species, like *S*. *aureus*	105
Oregano, Thyme, Cinnamon, Sweet fennel EOs	Cellulose acetate	Increased flexibility, Reduction in water vapor transmission rate	*Penicillum* spp.*, E. coli, S. aureus*	106
Pink pepper EO	Cellulose acetate		*S. aureus*, *L. monocytogenes* microbial growth decreased in sliced cheese	107
Rosemary EO	Cellulose acetate		Pathogenic microorganisms on chicken breast cuts	108
Rosemary EO, Aloe Vera	Cellulose acetate	Decreased tensile strength, water uptake and contact angle; increased hydrophobicity and free radical scavenger activity	*E. coli, B. subtilis*	109
Rosemary EO, Oregano EO	Cellulose acetate (electrospinning)		*E. coli, C. albicans* and *S. aureus* and anti‐biofilm effects	110
Thymol	Cellulose acetate	Reduced transparency, mechanical, OTR, and WVTR properties	*L. monocytogenes, S. aureus, E. coli, Pseudomonas aeruginosa, Klebsiella pneumoniae, and Salmonella enteritidis*	111
Thymol + organoclay	Cellulose acetate	Enhanced optical and mechanical properties	*Listeria innocua*	112
Thymol	Cellulose acetate (supercritical CO_2_ impregnation)	Decreased glass transition temperature; disappearing of crystalline structure	23 tested strains, in particular *S. aureus*	113
Thymol	Cellulose acetate	Anti‐adhesion surface properties	*P. aeruginosa, S. aureus*	114
Oregano EO + Montmorillonite clay	Cellulose acetate	increased oxygen and water vapor barrier properties, rigidity, thermal stability, and elongation	Phytopathogenic fungi: *Alternaria alternata, Geotrichum candidum, and Rhizopus stolonifer –* Postharvest conservation	115
Mustard EO	Cellulose sulfate (edible film)	Reduced TS, water sorption; increased elongation	*L. monocytogenes, E. coli, S. aureus, B. subtilis, A. niger*	116
Murta fruit extract	Methyl cellulose	Increased mechanical strength; decreased swelling index; affected thermal properties	*L. innocua*	117
Mexican Oregano EO	Carboxymethylated cellulose (CMC) ‐ (edible film)		*L. monocytogenes, S. aureus*	118
*Mentha spicata* EO	CMC, chitosan (edible film)		Increased strawberries shelf‐life; *L. monocytogenes*	119
*Mentha spicata* EO, *Ziziphora clinopodioides* EO	CMC (edible film)		Increased fresh and sauced chicken breast fillets shelf‐life; *C. jeuni*, *L. monocytogenes, S. aureus, E. coli, S. typhimurium*	120
Bay EO	CMC (edible film)	Increased antioxidant activity, WVP, UV‐light barrier effect;	*Escherichia coli, Candida glabrata*	17
*Zataria multiflora Boiss* EO	CMC (edible film)	Increased total phenol content, antioxidant activity; reduced transparency and solubility in water	*S. aureus, B. cereus*, *E. coli*	121
*Thymus daenensis* EO	Hydroxyl‐propyl‐methyl cellulose (edible film)	Reduced tensile strength and Young's modulus	Gram positive and Gram negative bacteria	122
*Mentha spicata* EO	Chitosan (edible coating)	Good appearance at sensory analysis	Increased fresh strawberries shelf‐life; *L. monocytogenes*	119
Red thyme and Oregano extracts	Chitosan (edible coating)		Increased fresh strawberries shelf‐life; antifungal activity	123
Rosemary essential oil	Chitosan	Decreased solubility, WVP and UV‐light transmission	*L. monocytogenes, Streptococcus agalactiae, E. coli*	124
*Ziziphora clinopodioides* EO	Chitosan (edible film)	Decreased solubility, WVP, UV‐light transmission, swelling index and TS	*L. monocytogenes, S. aureus, B. cereus*	125
Apricot (*Prunus armeniaca*) kernel EO	Chitosan	Decreased solubility, WVP and UV‐light transmission and transparency	*E. coli, B. subtilis*, inhibition of fungal growth on bread	126
Lemongrass EO	Chitosan	Increased elongation; decreased moisture content, WVP, solubility and TS	*B. cereus, E. coli, L. monocytogenes, Salmonella typhi*	127
Thyme EO	Chitosan (edible film)	Decreased water condensation in the head space of the packaging; good odor	Reduced yeast population – cooked ham	128
Thyme EO, Cinnamon EO, clove	Chitosan (edible film)	Increased moisture content, solubility in water, WVP, elongation at break. Opposite behavior for cinnamon EO	*L. monocytogenes, S aureus, Salmonella enteritidis, Ps. aeroginosa*	129
Cinnamon EO	Chitosan (edible film)	Decreased moisture content, solubility in water, WVP and elongation at break; Increased TS	*L. monocytogenes, Lactobacillus plantarum, Lactobacillus sakei, Pseudomonas fluorescens, E. coli*	31
EOs, gelatin	Chitosan	Increased UV‐light barrier properties, moisture absorption and WVP; Decreased transparency	*C. jejuni, E. coli, L. monocytogenes, Salmonella typhimurium*	130
*Cinnamomum zeylanicum* EO	Chitosan nanoparticles		Increased shelf‐life and physicochemical quality of cucumbers; *Phytophthora drechsleri*	131
*Zataria multiflora* EO	Chitosan nanoparticles		Protection of preharvest or postharvest fruit from decay – strawberries treatment; *Botrytis cinerea*	132
Carvacrol	Chitosan nanoparticles		Increased shelf life of fresh‐cut carrots	133
Cinnamon EO	Chitosan nanoparticles	Physicochemical quality maintained; sensory analysis	Increased shelf life of chilled pork; Psychrotrophic aerobic bacteria, lactic acid bacteria, Enterobacteriaceae	134
Frankincense EO	CMC/Chitosan biguanidine hydrochloride (edible film)	Decreased WVP, increased TS, EB;	*S. pneumonia, B. subtilis, E. coli*	135
Cinnamon EO	CMC/chitosan	Increased TS, WVP, EB and antioxidant properties; Decreased water solubility	*L. monocytogenes, P. aeruginosa*	136
Cinnamon and ginger EOs and oleic acid	CMC/chitosan	Increased EB; Decreased WVP	*A. niger*	137
Thyme, Oregano, Tea tree and Peppermint EOs	CNC/chitosan	Sensorial evaluation; Increased TS, EB, WVP	Increased shelf‐life of rice; *A. niger, Aspergillus flavus, Aspergillus parasiticus, Penicillum chrysogenum*	138
Thyme, Oregano EOs	CNC/methyl cellulose	Increased TS, EB, WVP	Increased shelf‐life of rice; *A. niger, A. flavus, A. parasiticus, P. chrysogenum*	139
Thyme EO	CNF/chitosan (edible coating)	Decreased weight loss, preserved anthocyanin content, better appearance	Sweet cherry storage	140
Oregano EO ‐ *Carum copticum* EO	CNF‐LCNF/chitosan	Increased water vapour barrier, water solubility and opacity; release controlling effect of CNF – LCNF	*E. coli, B. cereus*	,^141^, ^142^
Oregano EO	CNC/PLA	Increased EB; Decreased TS, TM;	*L. monocytogenes*; mixed vegetables	143
*Tanacetum balsamita* EO, propolis ethanolic extract	CNC/PLA	Increased TS, elastic modulus; Decreased elongation	*E. coli*,*B. cereus*,*S. aureus*,*S. Typhimurium*; vacuum‐packed cooked sausages	144
*Ziziphora clinopodioides* EO	CNC/PLA	No alteration of organoleptic properties	*Pseudomonas*spp.; Increased shelf‐life of minced beef	145
*Zataria multiflora* EO, propolis ethanolic extract	CNF gel/PLA	Increased WVP, TS, EM; Decreased transparency	*S. aureus*, *E. coli*, *Vibrio parahaemolyticus, L. monocytogenes*; Vacuum‐packed cooked sausages	146
Rosemary EO	Chitosan/PLA	Increased EM, TS; Decreased elongation; antioxidant activity; color change	Fresh minced chicken breast	147
Oregano, Cinnamon, Winter savory EOs	Alginate (edible film)		*Salmonella Typhimurium*, *L. monocytogenes*; ham slices	148
Cinnamon EO	CMC/sodium alginate	Increased WVP, oxygen permeability and elongation; Reduced moisture content and TS	*S. aureus; E. coli*; Bananas	149
*Savory*EO	Cellulose nanoparticles/Agar	Decreased TS, water solubility; increased EB, WVP, opacity;	*L. monocytogenes, B. cereus; S. aureus*	88
Summer savory EO	CMC/Agar	Increased mechanical flexibility, hydrophobicity; reduced transparency	*S. aureus*, *B. cereus*, *L. monocytogenes*, *E. coli*	150

### Incorporation of essential oils in the matrix

In active packaging applications, EOs are particularly interesting because they can be released as vapors from films, sterilizing both the headspace and the food surface. Moreover, they are approved by the FDA for food applications and are generally recognized as safe. So they are an attractive alternative to conventional antimicrobials, which have experienced a continuous increase in resistance from the microorganisms.^151^ Furthermore, while introducing a new agent inside the packaging material, not only the antimicrobial properties, but also the mechanical, barrier, and thermal properties of the final composite will be influenced and possibly improved.^9^, ^152^ As an example, the incorporation of EOs can reduce the water vapor permeability (WVP) of hydrophilic materials and can also decrease the tensile strength (TS), while increasing the elongation at break. This is possible thanks to the partial replacement of stronger polymer–polymer interactions by weaker polymer‐oil interactions in the film network.^21^ Similar results were obtained when different cellulose esters were tested as matrix for the incorporation of EOs. In fact, lemongrass, basil, and rosemary pepper EOs behaved in the matrix like plasticizers, affecting the Young's modulus, the tensile strength, and the elongation at break of the films.^153^

The incorporation of the EOs in a polymer matrix such as chitosan and nanocellulose could be done with different methods, which are chosen based on the materials characteristics and on the release kinetics requested by the final product. As an example, the active molecules or scavengers could be dispersed directly inside the matrix,^105^ or encapsulated^134^ inside a carrier prior to the addition in the packaging material. The latter procedure, indeed, allows more flexibility in terms of active substance dispersion in polymers that are not compatible and in terms of release rate control. In fact, microencapsulation increases the stability of these compounds and allows a controlled and continuous release to be achieved,^154^ which leads to the antimicrobial activity without altering the organoleptic properties of the food product.^155^ Essential oils can be microencapsulated into the matrix following several different technologies, such as spray drying, simple or complex coacervation, and extrusion.^154^ They are among the most effective techniques for protecting compounds against volatilization, oxidation, and thermal degradation.^156^, ^157^ Active ingredients could also be incorporated through a Pickering emulsion,^158^, ^159^ which stabilizes the oil‐in‐water solution interface by using solid particles,^160^ or through nano‐liposomal systems.^161^ Encapsulation not only protects the antimicrobial compounds from the effects of the outside environment^162^ but also allows the influence of the mechanical and transport properties of the film to be controlled.^163^, ^164^ On the other hand, direct application of EOs on the film could lead to inactivation of the antimicrobial compounds due to the interaction with the matrix and makes the control of its release difficult. For that reason, it is considered mainly for edible polymer films.^21^, ^165^, ^166^

While incorporating these active agents inside the matrix, it is essential to take into consideration that the concentration of the EOs’ active compounds depends on their origin. Different compounds could act against different microorganisms, so some EOs can be more active in certain types of food products. For this reason, mixing different EOs can be a strategy to widen their active spectrum and increase their effectiveness. Essential oils, indeed, often show synergistic interactions and, when used in combination, they show increased antimicrobial properties with respect to the single components.^167^ However, the antimicrobial activity of the EOs could decrease after dispersion in the polymer and the microbial population could vary depending on the food product, so *in vitro* and *in situ* analysis are usually required to define the real efficiency of the chosen solution.^20^, ^168^

A huge variety of essential oils and other plants extracts has been considered for this purpose.^169^, ^170^ However, oregano^171^, ^172^, ^173^, ^174^ and thyme^175^, ^176^ demonstrated the strongest antimicrobial effect due to the presence of the phenolic compounds thymol and carvacrol.^177^ These compounds have received substantial attention as useful natural antimicrobial agents. They exhibit a broad antimicrobial spectrum against different microorganisms^178^ and possess sufficient heat stability to withstand incorporation into packaging materials.^23^, ^179^

Due to these multiple possibilities, each active packaging system needs to be adapted based on the food product, not only in terms of the materials used but also in terms of mechanism of release and action. In fact, the antimicrobials present in the film could have a diffusive release in the headspace, with a decreasing effect over time, or act when in direct contact with the food, in case they are immobilized on the packaging surface or used as edible films.^10^, ^180^

## NANOCELLULOSE‐BASED ACTIVE PACKAGING

Cellulose is one of the most commonly used polymers for active and sustainable packaging production.^181^ Many different natural antimicrobial additives have been studied in combination with cellulose.^182^, ^183^, ^184^, ^185^ In the present section an overview of the latest applications in the field of active packaging based on essential oils (EOs) will be given. In particular, the focus will be on cellulose nanocrystals (CNC) and nanofibrils (CNF) and on nanocellulose derivatives such as cellulose esters (like cellulose acetate and sulfate) or cellulose ethers (such as methylcellulose and carboxymethylcellulose).

### Cellulose nanocrystals (CNC) and nanofibrils (CNF)

As mentioned above, nanocellulose crystals or fibrils, obtained from pristine cellulose fiber,^40^ have found several applications in the field of food packaging due to their unique properties. They are often used as fillers to improve the tensile strength of the composite material^50^ and the dispersion of the essential oils into the matrix.^116^ This type of solution will be discussed further below, while this section is more focused on the direct application of CNC and CNF as matrices to produce antimicrobial films.

In some cases nanocellulose has been used to stabilize and protect EOs. Souza *et al*., for example, obtained films with strong effects against *Bacillus subtilis* by preparing nanocellulose‐based Pickering emulsions with cinnamon essential oil.^103^ Zhou *et al*. analyzed the antimicrobial activity of oregano EO Pickering emulsion stabilized by CNC.^102^ Good stability at higher CNCs concentration and pH values, or at lower oil/water ratio and salt concentration was demonstrated, together with a slightly higher antimicrobial effect. The OEO Pickering emulsion exhibited an inhibitory effect against *Staphylococcus aureus*, *Saccharomyces cerevisiae*, and *E. coli*, with a minimum inhibition concentration (MIC) of 12.5 μL mL^−1^. The highest antimicrobial effect was obtained against *B. subtilis*, with an MIC of 6.25 μL mL^−1^.

The direct incorporation of ginger EO and citric acid in CNF edible coatings (20 g kg^−1^ and 10 g kg^−1^, respectively) was instead studied by Khaledian *et al*. and increased the meat shelf life up to 6 days.^104^ The combined effect of the EO and citric acid resulted in an increase of the antimicrobial properties and the overall acceptability of the food samples. The antimicrobial activity of cellulosic pads amended with oregano (OEO), thyme (TEO) and cinnamon (CEO) EOs was demonstrated.^105^ They were effective against meat bacterial species and other common foodborne pathogens like *S. aureus*.

Direct incorporation of the essential oils' active compounds was also considered for the addition of polyphenols in a cellulose dispersion obtained through mechanical fibrillation. The resulting films had low porosity and high compactness; they thus possessed good barrier properties and improved hydrophobicity.^186^ The addition of tannin to CNF reduced the air permeability more than six times in comparison with pure film, reaching a value of 3.1 mL min^−1^, which is comparable to the polypropylene one. The release of tannins also ensured antioxidant activity for 48 h. These films were thermally stable until ca. 230 °C and chemically resistant against common organic solvents. A possible application could be for dried food packaging (like rice and pasta), and for preserved fruits, vegetables, and meat.

Direct incorporation, therefore, leads to a very viable and simple method to produce nanocellulose‐based films. Indeed, the antimicrobial activity does not seem to be reduced by the interaction between the fibers and the EOs. This, however, is not the only option available and other methods were used with good results. For example, microencapsulation is a common technique used to protect the sensitive compounds inside a carrier. For example, Saini *et al*. studied the microencapsulation of carvacrol in beta‐cyclodextrin (*β*‐CD), directly grafted on the carboxyl groups of TEMPO‐oxidized CNF.^187^ These films showed a sustained release of the active molecule over 150 h and then reached an equilibrium in water. Moreover, carvacrol antimicrobial activity against *B. subtilis* was increased by the presence of *β*‐CD from 3 to 50 h. This is due to the three‐dimensional shape of the *β*‐CD, which forms an inner hydrophobic cavity with an outer hydrophilic wall.^188^ In this way it can entrap molecules and create complexes either by hydrogen bonds, or hydrophobic or Van der Waals interactions. The release rate of the included molecules is thus strongly influenced by those interactions.^189^

### Cellulose esters

In the field of active packaging, the most studied cellulose esters resulted to be cellulose acetate (CA)^190^ and cellulose sulfate (CS).^116^ Cellulose acetate is produced, replacing the hydroxyl groups from the cellulose backbone chain with acetate groups (Fig. 3(c)) by means of a reaction of native cellulose with acetic anhydride.^115^ While CS is obtained by partially or completely substituting the hydroxyl groups of the nanocellulose by sulfate groups (SO_3_^−^; Fig. 3(d)).^191^

Several EOs – oregano (OEO), cinnamon (CEO), and sweet fennel – were incorporated in CA films in different combinations to test the antimicrobial effectiveness of the biodegradable composite.^106^ Oregano essential oil combined with CEO showed good results in terms of reduction of water vapor transmission rate and antimicrobial activity against *Penicillum* spp. and *E. coli*, with diameters of inhibition zones of 2.74 and 1.14 cm, respectively. Films incorporated with pure OEO were more effective against *S. aureus*, with 3.75 cm of inhibition zone. All the other combinations of EOs were effective against these microorganisms but with a much lower inhibition zone.

The effect of pink pepper EO (PPEO) in CA was also studied,^107^ with films active against *S. aureus* and *Listeria monocytogenes*. The antimicrobial effect started from a concentration of 20 g kg^−1^ of PPEO, which demonstrated the capacity to diffuse in solid, liquid, and gas phase thus reaching the contaminated cheese used for tests. Cellulose acetate active films incorporated with rosemary EO were produced to control the pathogenic microorganisms on chicken breast cuts.^108^ The films showed increasing antimicrobial activity at increasing EO concentration. However, it was necessary to reach a concentration of 500 g kg^−1^ to obtain an effective result. The effect of rosemary and aloe vera EOs on CA films was studied.^109^ The presence of these EOs decreased the tensile strength, the water uptake, and the contact angle, but increased the free radical scavenging activity. The antimicrobial activity against *E. coli* and *B. subtilis* increased as the percentage of rosemary and aloe vera oil increased in CA membranes. In particular, the films containing 800 mL kg^−1^ of EOs (based on CA weight) showed no bacterial growth over 7 days of storage. Moreover, the electrospinning technique has been used to create CA nanofibers with 1 and 5 mL L^−1^ of rosemary and oregano EOs.^110^ The fibers with 5 mL L^−1^ of oregano EO showed the best antimicrobial and anti‐biofilm effects, especially for *E. coli* and *Candida albicans*.

Harini and Sukumar studied the direct incorporation of thymol, the major active compound present in the polar fraction of Oregano EO, inside CA.^111^ The films were produced by vacuum drying and the difference between bulk dispersion and surface immobilization of the active compound was studied. The films that were obtained were transparent and showed, respectively, >90% and ca. 65% thymol retention. The UV‐assisted surface immobilization decreased the mechanical and barrier properties of the CA films. Good antioxidant and antimicrobial properties were obtained in general, even if films with the thymol dispersed in the bulk showed higher activity. These films were active against *L. monocytogenes, S. aureus, E. coli, P. aeruginosa, Klebsiella pneumoniae*, and *Salmonella enteritidis* with a minimum inhibition concentration of 20 mg L^−1^. The effect of thymol was also studied when used together with organoclay, which increased the antimicrobial effect against *Listeria innocua*.^112^

The release of thymol from a cellulose acetate film impregnated using supercritical carbon dioxide (scCO_2_) has been studied.^113^ This technique was already studied using CA^192^ and other polymers^193^ and it has already been used on an industrial scale for the extraction of low volatility and/or thermal sensitive compounds. Due to its low viscosity and surface tension, scCO_2_ can easily penetrate into a solid matrix. This facilitates the impregnation process and ensures a good distribution of the active molecules.^114^ The CA structure and morphology of the films obtained in this way depended on the thymol content. Increasing the thymol content above 137 g kg^−1^ led to a decrease in the glass transition temperature up to 29 °C, and the crystalline arrangement of the CA disappeared. In general, the thymol release, which depends on the concentration and on the release medium, required up to 3 days. Thymol was detected on the CA surface as well, thus allowing antimicrobial activity through direct contact. The impregnated CA showed antibacterial activity against 23 tested strains, in particular against methicillin‐resistant *S. aureus*, a cause of fatal infections in animals and humans.^113^ The optimal thymol loading for an efficient reduction of biofilm formation was in the range from 260 to 300 g kg^−1^.^114^ In particular, the film containing 300 g kg^−1^ of thymol exhibited anti‐adhesion properties on its surface. They were active against all tested strains, including antibiotic‐resistant *Pseudomonas aeruginosa* DM50 and methicillin‐resistant *S. aureus*. Furthermore, CA incorporated with OEO and montmorillonite clay were used to control the growth of phytopathogenic fungi.^115^ It was demonstrated that contemporary addition of active oils and nanoclays allowed active films to be obtained with a decreased water vapor transmission rate and improved thermal stability.

Cellulose sulfate was also investigated as a potential matrix for the development of food packaging films, although to a lesser extent with respect to CA.^191^ Cellulose sulfate‐based films with slow release of mustard EO due to the presence of *β*‐CD were tested.^116^ The mustard essential oil (MEO) had already been studied as active agent in edible films against *L. monocytogenes*.^194^ The addition of MEO to CS reduced the TS and the water sorption without affecting the WVP and increased the elongation at break (EB). The films also showed strong antimicrobial activity against *E. coli, S. aureus* and modest activity against *B. subtilis* and *Aspergillus niger*, which was attributed to the MEO.

### Cellulose ethers

Etherification is a widely used chemical pretreatment method that facilitates cellulose defibrillation to prepare CNF. Cellulose ethers can be obtained by a first activation of the fibers with an aqueous alkali hydroxide, such as NaOH, and then converting the hydroxyl groups to carboxymethyl moieties.^28^

In the field of active packaging, the functionality of biocomposite based on methyl cellulose and carboxymethyl cellulose in combination with plant extracts, such as murta fruit (*Ugni molinae*) or curcumin^195^ was investigated.^117^ The mechanical strength and water vapor barrier properties of the films resulted improved, as well as the antioxidant and antimicrobial activity.

Studies on carboxymethyl cellulose (CMC) were conducted by incorporating Mexican OEO at different values of pH.^118^ It was found that antimicrobial treatment against *L. monocytogenes* and *S. aureus* were more effective at lower pH values (pH = 5 and 2.5 g kg^−1^ of Mexican OEO). The *Mentha spicata* EO (MSEO) was also investigated.^119^ The treatment of fresh strawberries with CMC with 2 g kg^−1^ MSEO resulted in a decrease of *L. monocytogenes* population, while physicochemical and organoleptic properties were maintained. Moreover, MSEO was used together with *Ziziphora clinopodioides* EO (ZiEO) to create CMC active coatings for the extension of fresh and sauced chicken breast fillets shelf life.^120^ In fact, the application of CMC with ZiEO (2.5–5 g kg^−1^) and MSEO 5 g kg^−1^ increased the shelf‐life up to 14 days and completely inhibited the growth of *Campylobacter jejuni*, while the growth of *L. monocytogenes, S. aureus, E. coli*, and *Salmonella typhimurium* was retarded.

**Figure 5:**
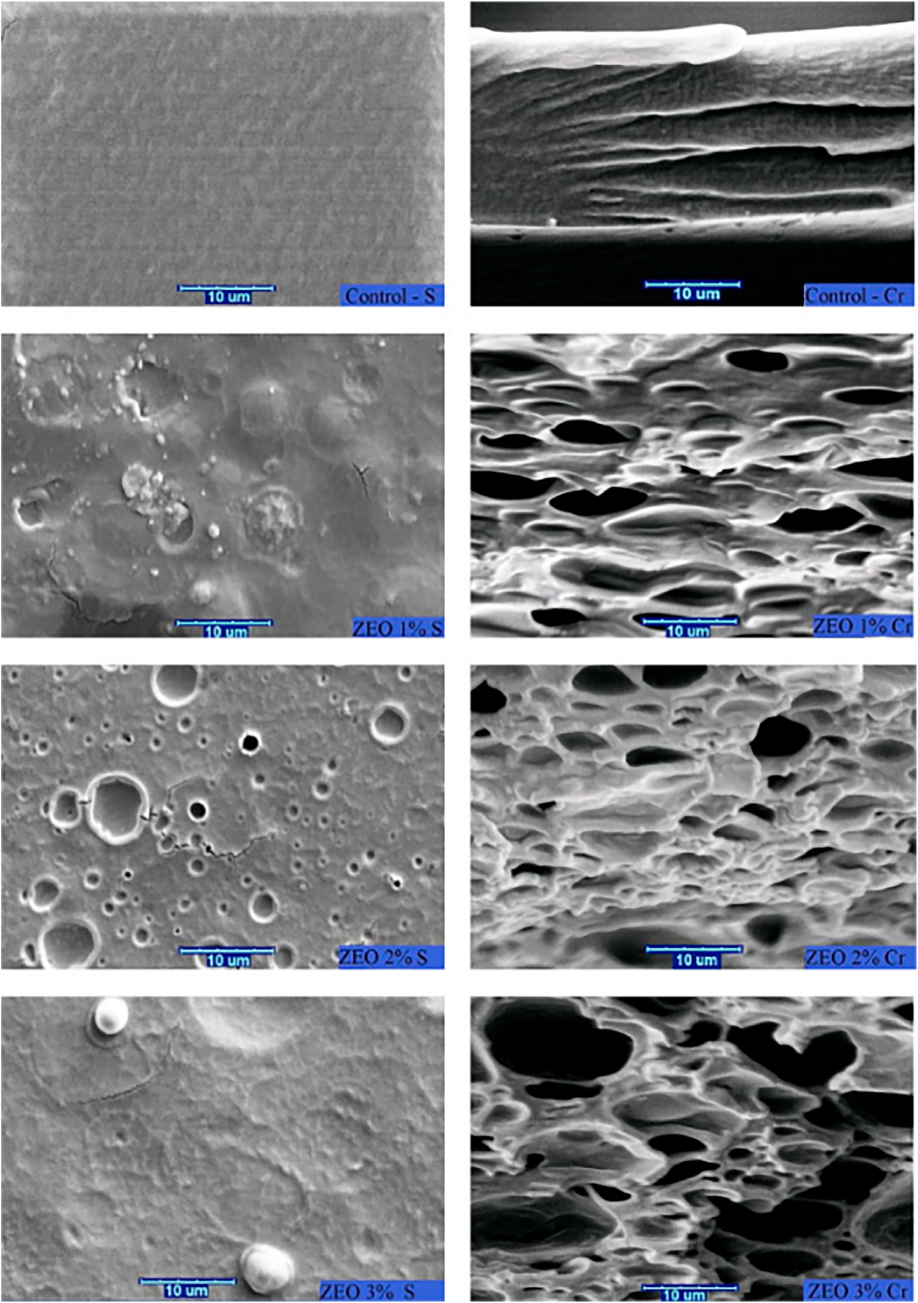
Scanning electron microscopy images of the surfaces (left) and cross‐sections (right) of CMC films containing different ZaEO concentrations (from the top: control, 10, 20 and 30 g kg^−1^ of content).^121^

The effect of bay EO on CMC was also considered,^17^ and high antioxidant activity (up to 99%) and, inhibition of microorganism growth (*E. coli* and *Candida glabrata*) were reported. Good barrier properties against water vapor (50% improvement with respect to CMC in films containing 150 g kg^−1^ of EO) were observed and the UV‐light barrier effect was increased (almost 100% of protection). Higher water solubility (93%) was finally found, which ensured material biodegradability. Carboxymethyl cellulose was also incorporated with *Zataria multiflora Boiss* EO (ZaEO).^121^ The increase in the ZaEO content led to a decrease in transparency and an increase in total phenol content and antioxidant activity. Figure 5 shows the SEM images of the surface (left) and cross‐sections (right) of CMC films containing different concentrations of ZaEO (10, 20, and 30 mL L^−1^). Pure CMC film appeared homogeneous and smooth, whereas the presence of ZaEO led to a more heterogeneous structure. The porous structure could be due to the evaporation of the EO during drying or to the entrapped air bubbles during the fabrication of the membranes. The films with the highest ZaEO content (30 mL L^−1^) had the best essential oil dispersion in the matrix. They also showed the highest microbial inhibition, in particular against *S. aureus, B. cereus*, and *E. coli*. Raeisi and coworkers added grape seed extract (GSE) to the same composite the and observed the effect on the shelf life of rainbow trout fillets.^196^ The minimum number of total viable bacteria (lactic acid bacteria and *Pseudomonas* spp.) was determined in the fillets coated with CMC plus 20 mL L^−1^ ZaEO and 10 mL L^−1^ GSE. The fillets containing 10 mL L^−1^ of both compounds had the best organoleptic properties.

Edible films containing *Thymus daenensis* EO from wild and cultivated plants, loaded in hydroxyl propyl methyl cellulose (HPMC), were also produced.^122^ The uniform incorporation of the nanoemulsions into the matrix led to a plasticizing effect and an antimicrobial effect against several microorganisms. In particular, the EO from the wild plant showed better antimicrobial activity against gram‐positive bacteria, whereas the EO from the cultivated plant was more effective against gram‐negative ones. This difference is due to the various quantities of components in the EOs: wild EO contained smaller amounts of thymol and carvacrol but was richer in *ρ*‐cymene. The latter is a precursor of carvacrol, and it was found to have a synergistic effect. In fact, when combined, these two substances can cause swelling of the cytoplasmic membrane.^23^

The literature reviewed in this section confirms that cellulose‐based matrixes with EOS added are a promising green alternative for food active packaging applications. Almost all the works cited demonstrated its effectiveness against gram‐positive and gram‐negative bacteria, irrespective of the method used for coupling. Only a fraction (about 30%) of them, however, tried to implement the developed solution on real packaging systems, mostly on cheese and meat products, and only 10% also focused on complete sensory evaluation. While mechanical properties were often analyzed and were only partially affected by the addition of EOs, there is still a need for further research to completely understand the potential of active packaging based on the coupling of EOs and nanocellulose and its derivatives. Almost half of the ideas developed in the field focus on edible films, pushing the concept of packaging itself to change from the consumer's viewpoint.

## CHITOSAN‐BASED ACTIVE PACKAGING

Chitosan, as previously explained, has been demonstrated to be suitable and convenient for development as a novel food packaging system due to its intrinsic antimicrobial activity and its film‐forming ability.^59^, ^60^ The mechanical and barrier properties of chitosan have also been largely reviewed and compared with those of synthetic plastics.^197^ The most important works regarding chitosan in packaging applications are related to antibacterial packaging based on the materials’ intrinsic properties^97^ or on their synergetic effect with other active substances.^198^, ^199^, ^200^ In the literature it is possible to find many studies on the incorporation of natural antimicrobials such as nisin,^199^, ^201^ polyphenols,^202^, ^203^, ^204^ and various plant extracts.^63^, ^205^, ^206^, ^207^, ^208^, ^209^, ^210^, ^211^ These advances are related to the use of chitosan‐based materials in various fields such as wound healing, food packaging, and the textile and biomedical sectors.^212^ In the following paragraph, only the works on the incorporation of essential oils for packaging application will be analyzed. In particular, the application will be divided considering the production of active materials in the form of films or in the form of nanoparticles to produce active (edible) coatings.

### Active chitosan films

As in the case of nanocellulose, most of the studies involving chitosan/EOs active packaging were focused on the analysis of antimicrobial activity on different gram‐positive and gram‐negative pathogens. The influence on the physical and mechanical properties of the films was also studied. As an example, the incorporation of rosemary EO up to 15 mL L^−1^ was able to produce chitosan films with antimicrobial activity against *L. monocytogenes*, *Streptococcus agalactiae* and *E. coli*. The treatment also decreased their light transmission in UV light and the water uptake by about 25% and 85%, respectively.^124^ In other works, fennel, peppermint,^213^ and *Citrus limonia* ^214^ essential oils were tested, showing that they also help in decreasing the moisture content and protect from UV light. The same trends were observed with the use of *Ziziphora clinopodioides* EO, red grape seed extract,^125^ and with the use of apricot (*Prunus armeniaca*) kernel EO.^126^ The incorporation of lemongrass essential oil had similar results, with a 101% improvement in the elongation at break and 15% reduction in water vapor permeability.^127^ Other authors tested thyme EO in chitosan and found it more effective as antimicrobial than clove and cinnamon EOs.^129^ Moreover, the presence of thyme and clove EOs in chitosan films led to an increase in the moisture content (+23%), solubility in water (+28%), the water vapor transmission rate (0.004 g s^−1^ m^−1^ ^2^), and finally elongation at break (+34%). Interestingly, cinnamon‐enriched films showed the opposite behavior, with a decrease of EB and an increased tensile strength. The same trend was also observed by other authors, which suggested that these results are due to the cross‐linking effect of CEO components within the chitosan matrix.^31^ However, the antimicrobial effect against several gram‐positive bacteria (*L. monocytogenes, Lactobacillus plantarum, Lactobacillus sakei*) and gram‐negative bacteria (*Pseudomonas fluorescens, E. coli*) was satisfactory for a concentration of 20 mL L^−1^ of EO.

Chitosan films enriched with essential oils have recently been amended with gelatin and characterized.^130^ They were active against *C. jejuni, E. coli, L. monocytogenes* and *S. typhimurium*. They also showed good barrier properties against UV light and an increase in moisture absorption and water vapor permeability. Chitosan films were also used in combination with propolis extract instead of essential oils. As the extract has a high polyphenols content, the antimicrobial and mechanical improvements observed were similar to those obtained with essential oils.^215^

**Figure 6:**
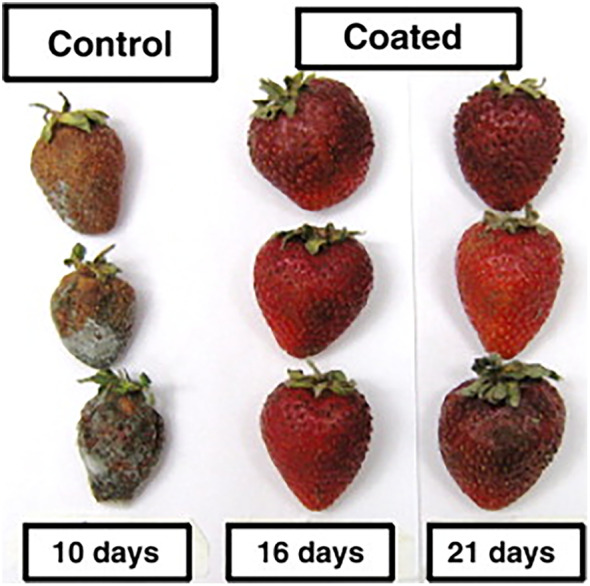
Appearance of strawberries coated with modified chitosan‐based formulation during the shelf‐life test. The control (on the left) after 10 days shows worst conditions compared with coated strawberries after 21 days (on the right).^123^

With regard to direct application on food products, fresh strawberries are among the most common food used to test antimicrobial properties in active packaging. Their treatment with chitosan and *Mentha spicata* EO 2 g kg^−1^ resulted in a decrease in the *L. monocytogenes* population while physicochemical and organoleptic properties were maintained.^119^ Fresh strawberries were also treated with edible bioactive chitosan films containing red thyme and oregano extracts, substantially increasing the shelf‐life, as visible in Fig. 6.^123^ Thyme EO was also tested as an antimicrobial agent inside a chitosan matrix used for cooked ham packaging. Its presence reduced the water condensation inside the package and the odor was perceived as desirable in the food product. Moreover, the yeast population was reduced by the antimicrobial agent, while the aerobic mesophilic bacteria, the lactic acid bacteria, and the enterobacteria were not affected.^128^

### Chitosan nanoparticles

In addition to its function as matrix, chitosan has also been considered for the production of nanoparticles (CSNs),^216^ alone or in combination with nanoclays^217^ and nanofibers.^218^ They can be mixed homogenously with different essential oils to enhance the shelf life of various food products. This procedure is usually applied to fruit or vegetables, which are dipped in the coating solutions containing the EOs entrapped into the chitosan nanoparticles. The effect of chitosan nanoparticles incorporated with *Cinnamomum zeylanicum* EO on the cold storage of cucumber was observed by Mohammadi *et al*.^131^ The CEO encapsulated by CSNs provided a better antimicrobial activity against *Phytophthora drechsleri*, improving the shelf‐life and the physicochemical quality of the cucumbers. The same research group also encapsulated *Zataria multiflora* EO in chitosan nanoparticles through ionic gelation. They studied the release rate and the performance *in vitro* and *in vivo* against *Botrytis cinerea*, the major cause of gray mold disease.^132^ The findings revealed a promising technique to prevent postharvest fruit from decay and extend the storage life, without the use of synthetic fungicides. Carvacrol‐loaded chitosan nanoparticles were also tested to maintain the quality of fresh‐cut carrots.^133^ The vegetables dipped in the washing treatments presented a 2–6 lower log CFU g^−1^ units in comparison with control samples and better sensory and physicochemical quality after 13 days at 5 °C. Similarly, the effect of cinnamon EO in chitosan nanoparticles on the conservation of chilled pork was studied.^134^ In this case, the food product was wrapped with low‐density polyethylene films, whose inner surface was coated with layers of chitosan nanoparticles of different sizes, loaded with the EO. After 15 days of storage at 4 °C, a significant decrease in microbial growth, pH, and peroxide concentration was observed for films containing microparticles (527 nm).

It can be concluded that the incorporation of various essential oils in different concentrations can be very effective for the shelf‐life elongation in fresh food products like vegetables and meat. In fact, these active components not only allowed the reduction of microbial growth but also showed a decrease in water vapor permeability of the films, together with an increase in thermal stability and elongation at break. Interestingly, in comparison with nanocellulose in the case of chitosan‐based active packaging, there was a higher number of works (about 60% of the articles reviewed) dealing with direct application on food products. They were mostly vegetable products or fruit, but some attention had also been given to meat products and bakery. The same percentage focused also on analyzing the impact of essential oils and active agents' incorporation on the final mechanical properties of the film, but once again only a small percentage (~20%) also included a comprehensive sensory evaluation of the packaged food.

## BIOCOMPOSITE FILMS

Bio‐nanocomposite materials consist of a matrix composed by a bio‐based polymer and a dispersed nanometric phase, which is meant to improve the properties of the base material.

The majority of nanocomposite materials are focused on the improvement of the mechanical and structural properties of the matrix. It is quite common, for example, to use cellulose nanowhiskers or cellulose nanocrystals as reinforcements, due to their high tensile strength and modulus.^29^ They can be easily dispersed in hydrophilic polymers,^55^ and can be modified to increase the compatibility with non‐polar matrixes, improving their mechanical, physical, thermal, and optical properties. At the same time they help the homogeneous homogenous dispersion of the active molecule previously integrated.^29^ The contemporary incorporation of nanoparticles and essential oils has therefore become a common technique to endow the final biocomposite materials with additional antimicrobial properties while maintaining sufficient mechanical strength.^219^ The incorporation of essential oils in nanocomposites also allows the modification of properties not directly related to the antioxidant and antimicrobial activity, such as film transparency or hydrophobicity.

Nanoparticles can be treated or coupled with the EOs before or after the addition to the matrix. Several techniques have been used to create biocomposite films, depending on the type of materials involved and the final application of the composite. Some of the most used are, for example, extrusion, solvent casting, impregnation, layer‐by‐layer deposition and spin‐coating.^50^ Among the several existing techniques, then, the creation of nanoemulsions of the EOs and nanoparticles prior to dispersion in the active matrix represents a step forward for the food packaging applications, and in particular for the incorporation of active compounds in films and coatings.^220^

### Chitosan‐cellulose composites

Biocomposite films where chitosan and cellulose in different forms are used together as matrix and/or reinforcement for active food packaging applications are rather common in the scientific literature.^221^, ^222^, ^223^ The focus, in this concern, is not only on the antimicrobial activity, but also on the influence that the different components have on the mechanical properties of the film. In this case, *in vitro* studies were most often conducted, without direct testing on food products.

For example, CNC incorporation in chitosan matrix allowed to obtain active films which resulted to increase chicken meat shelf‐life.^223^ Many works on their combination with other polymers and with agricultural or food process wastes have been reported. For example, the production of biodegradable nanocomposites from carrot minimal processing waste (CMPW) was optimized by adding high‐pressure microfluidized cellulose fibers as mechanical reinforcement.^224^ While a composite film based on bacterial cellulose, chitosan and curcumin has been recently developed,^225^ considering also the case of multi‐nanofiber system by incorporating in the matrix both cellulose and chitin nanofibers.^226^ The nanofibers allowed a good dispersion of curcumin nanoparticles in the films, which reduced the TS and increased the WVP. However, these modifications were counterbalanced by the presence of chitin nanofibers, which positively affected both mechanical and barrier properties of the material. The films that were produced also showed antioxidant and antibacterial activity against *E. coli* and *S. aureus* with inhibition ratios of 65% and 75%, respectively. Conversely, the incorporation of curcumin extract in pure chitosan resulted in the opposite effect on mechanical properties.^227^ This indicates that, even if these materials have certain properties and known effects on the matrix, their combination strongly influences the final properties of the composite.

Moreover, binary edible films made from CMC and chitosan biguanidine hydrochloride (CGg) activated with frankincense oil (FO) or titanium oxide nanoparticles^228^ were prepared and analyzed by Salama *et al*.^135^ The presence of FO resulted in a lower WVP, higher TS and EB, without any change in transparency. These films exhibited antibacterial activity against *S. pneumonia, B. subtilis*, and *E. coli*. The same biopolymers were used to produce chitosan/CMC films incorporating glutaraldehyde, cinnamon EO, and oleic acid (OA), in order to study their simultaneous effect.^136^ In fact, cross‐linkage by glutaraldehyde improved the mechanical properties, and its use together with the CEO increased the film's bioactivity. The presence of OA also increased the antimicrobial and antioxidant activity. The inclusion of both CEO and OA significantly increased the WVP, due to significant changes in the microstructure of the biocomposite. This could be due to the covalent interactions between the essential oil constituents and/or OA with the biopolymer chains.

Similar results were found when investigating the effect of cinnamon and ginger EOs on chitosan/CMC films emulsified with oleic acid.^137^ Clear differences appeared between cinnamon EO and ginger EO incorporated films. As the amount of each essential oil increased, the crystallinity decreased with the former essential oil, while it increased with the latter one. In fact, the cinnamaldehyde present in cinnamon could interact with the network created by CMC, chitosan and oleic acid, acting as plasticizer and inhibiting close packing in the polymer chains. Moreover, the cinnamon‐incorporated films showed higher antifungal activity *in vitro* against *A. niger* and a greater increase in elongation at break percentage: +328% compared to +111% of the ginger films.

Chitosan‐based films reinforced with CNCs and encapsulating thyme, oregano, tea tree, and peppermint EO nanoemulsions showed improved mechanical properties and better release of the active compound.^138^ They were tested *in vitro* and *in situ* against *Aspergillus niger, Aspergillus flavus, Aspergillus parasiticus*, and *Penicillum chrysogenum*, reducing their growth by 51–77%. An *in situ* experiment on inoculated rice during 8 weeks of storage resulted in a 2 log reduction of the fungal growth. The irradiation of the materials with a dose of 750 Gy of ionizing radiation further increased the antifungal and mechanical properties. The same research group also considered methyl cellulose (MC) reinforced with CNC and amended with a blend of oregano and thyme EOs.^139^ The optimal conditions were found to be 75 g kg^−1^ CNC into MC containing 5–7.5 g kg^−1^ EO. The films exhibited the same antifungal activity as the previous study^138^ and the irradiation treatment resulted again in improved antifungal and mechanical properties. The presence of different matrices and EO concentrations in the two cited works led to different characteristics in the final films. The chitosan‐based and the CM‐based films treated with irradiation had a tensile strength of 57 and 64 MPa, respectively – an increase of the elongation at break of 36% and 26% and an increase in water vapor permeability of 24% and 5%. Chitosan films enriched with cellulose nanoparticles were also studied in combination with ethanolic propolis extract.^229^

Chitosan matrix incorporated with 10 g kg^−1^ nanocellulose fiber and 10 g kg^−1^ thyme EO was tested on sweet cherry quality during storage.^140^ After 5 weeks of fruit storage within the edible coating, the nanocomposite affected the fruit's water retention, decreasing the weight loss and preserving the anthocyanin content. Moreover, the total sugar content increase indicates dehydration and decomposition of organic acids in the fruit during the storage time.

The effect of cellulose and lignocellulose nanofibers (LCNF) (40 g kg^−1^) as nanoreinforcement on *Origanum vulgare* EO‐loaded chitosan films (50 g kg^−1^) was investigated.^141^ The films without nanoreinforcement, containing only the EO, showed higher antioxidant and antimicrobial activity against *E. coli* and *B. cereus* than bionanocomposite films, where the release controlling effect of CNF and LCNF is present. The incorporation of EO and CNF/LCNF, on the other hand, improved solubility and the water vapor barrier but affected the color properties. This is due to the new hydrogen bonds created between the chitosan chains, the EO, and the nanofibers. Lignocellulose nanofibers resulted to better disperse the EO into the chitosan matrix, which led to better properties of the composite. The same investigation was also conducted with the use of *Carum copticum* EO, which gave similar results with respect to the previous study.^142^

### Composites involving other biopolymers

In this paragraph, relevant studies regarding the use of biopolymers other than nanocellulose and chitosan as a matrix for biocomposite materials for active food packaging are reported. The most studied in the recent years are PLA, sodium alginate, and agar films, as already explained in the ‘active packaging biopolymers’ paragraph.^230^ Many other studies regarding biocomposite films for active packaging based on different biopolymers such as whey protein isolate,^231^ starch,^232^ and others^230^ could be found in the literature. However, most of them are not based on the use of essential oils and therefore will not be considered in the context of this review.

#### Combination of PLA and nanocellulose or chitosan

Poly‐lactic acid‐cellulose nano crystal (PLA‐CNC) nanocomposite films containing OEO were tested against *Listeria monocytogenes* in mixed vegetables.^143^ It was observed that the presence of the OEO did not affect the WVP, but increased the EB while reducing the TS and the tensile modulus. These films demonstrated strong antimicrobial activity through the continuous release of phenolic compounds over the tested period.

The fabrication of a PLA composite with the addition of CNC is expected to increase the mechanical properties of the material significantly. Interestingly, however, Khodayari *et al*. showed that such improvement was more pronounced when it was coupled with different concentrations of *Tanacetum balsamita* EO (TBE) and propolis ethanolic extract (PEE).^144^ While PLA films containing PEE, alone or coupled with CNC, could not inhibit the growth of bacteria, the presence of TBE allowed gram‐positive and gram‐negative bacteria to be affected, especially *B. cereus*. All films containing TBE showed significant antibacterial effects against aerobic mesophilic bacteria, lactic acid bacteria and psychrotroph. The same nanocomposite was created using *Ziziphora clinopodioides* EO to improve beef meat shelf life.^145^ The microbial population after 11 days of storage of minced beef decreased 1 to 3 log CFU g^−1^ and films containing 20 g kg^−1^ EO extended the shelf‐life without any alteration of the organoleptic properties.

Poly‐lactic acid was also used in combination with CNF gel, through the incorporation of *Zataria multiflora* essential oil (ZaEO) and propolis ethanolic extract (PEE), by the solvent casting method.^146^ The gel was obtained directly from wood particles by a mechanical method. The addition of ZaEO and PEE made the films more flexible. The presence of CNF improved the WVP, the TS (+32%) and the elastic modulus (+19%). The maximum antibacterial effect was recorded in the film containing both ZaEO (5 mL L^−1^) and PEE. In particular, the PLA/10 g kg^−1^ of ZaEO/PEE composite was able to increase the shelf life of sausages up to 40 days, addressing the antimicrobial activity against *S. aureus, E. coli, Vibrio parahaemolyticus* and *L. monocytogenes*.

Poly‐lactic acid has also been combined with chitosan‐based materials. For example, Fiore *et al*. tried to coat PLA film with chitosan enriched with rosemary essential oil for the development of fresh minced chicken breast application.^147^ With a concentration of 20 g kg^−1^ of essential oil in the coating it was possible to reduce by 25% the water vapor permeability and increase the antioxidant activity. In general, those films demonstrate the ability to improve the shelf‐life of fresh meat products.

#### Alginate

Alginate edible films were incorporated with oregano, cinnamon or winter savory EOs and their antimicrobial activity was tested against *Salmonella typhimurium* or *Listeria monocytogenes* in ham slices.^148^ The films were pretreated with different concentrations of calcium chloride. In fact, the formation of ionic bonds produced an insoluble gel, which affected the release rate of the active compounds. Cinnamon‐based film pretreated by immersion in a 20% CaCl_2_ solution was the most effective against both pathogens.

The application of sodium alginate (SA) films activated with CMC, CEO as antimicrobial agent, glycerol as plasticizer, and Tween® 80 as surfactant were studied by Hal *et al*.^149^ At the highest CEO concentration of 15 g L^−1^, the inhibitory effect against *S. aureus* increased with the increase in the Tween® 80 concentration. The incorporation of Tween® 80 in the SA/CMC matrix may facilitate the release of CEO from the film matrix and promote its diffusion into the surroundings, thus increasing the antimicrobial activity of the film. These film‐forming solutions were coated on banana fruits to test possible shelf‐life extension. The bananas coated with SA/CMC containing 15 g L^−1^ CEO deteriorated more rapidly than those with control coating. This was probably caused by the increase in oxygen permeability of the films, which, associated with the higher oxygen solubility in CEO, favored the oxidation of phenolic compounds. Water content, on the other hand, was decreased in the presence of CEO and, due to the increased hydrophobicity of the films, WVP was also reduced for SA/CMC films incorporated with 15 g L^−1^ CEO. Concerning the mechanical properties, finally, it is interesting to notice that the effects of Cinnamon EO are different from the ones observed in other matrices such as chitosan.^129^ Due to the specific interactions between the fillers and the matrix itself, the presence of CEO led in this case to the increase of EB and the decrease of TS of the films.

#### Agar

Another example of biocomposite used in active packaging applications is the agar film reinforced with cellulose nanoparticles in presence of *Savory* EO (SEO) studied by Atef *et al*.^88^ The addition of SEO decreased tensile strength, Young's modulus, and water solubility, while increasing the elongation, WVP, and opacity of the nanocomposite film. In addition to these changes, the agar/cellulose‐based nanocomposite showed noticeable antimicrobial activity. In particular, the film containing 15 g kg^−1^ SEO demonstrated the highest inhibition zone, especially against *L. monocytogenes* and *B. cereus*, while *E. coli* was more resistant. To improve the mechanical flexibility and the thermal stability of agar, summer savory EO was incorporated into a CMC‐agar film.^150^ The biocomposite showed good inhibition against gram‐positive bacteria and an improvement in mechanical flexibility and hydrophobicity, at the price of a reduced transparency. The same biocomposite was created with grapefruit seed extract. An increase in UV barrier properties, moisture content, water solubility, and water vapor permeability was observed, with a decrease in tensile strength, elastic modulus, and surface hydrophobicity^93^, ^233^.

As a conclusion, after reviewing the research papers on biocomposite films for active packaging, the same trends observed for pure nanocellulose and chitosan are confirmed. In fact, the great majority of the reviewed studies (about 80%) reported *in vitro* tests against several types of microorganisms, coupled with analysis of the materials’ mechanical properties. This was necessary to characterize completely more complex materials involving the presence of different substances and to clarify the different effect of EOs chitosan and nanocellulose fibers on the final materials. Interestingly, however, a consistent proportion (above 60%) of the works reported also tested the films on food products, primarily on meat (sausages and ham) but also on vegetables and rice.

## CONCLUSIONS

In recent years research on packaging materials has been boosted strongly by the need to increase the sustainability of packaging while further reducing food spoilage. This has attracted much attention to renewable materials, such as chitosan and cellulose, and the use of natural compounds, such as essential oils, to impart antimicrobial features to the package, thus increasing the fresh product's shelf life.

This review has focused on this application, trying to report the use of the materials, alone or in combination with other biopolymers (such as PLA, agar and alginate), to obtain a completely renewable active packaging solution. Solutions involving the use of essential oils were considered due to the interest in such natural antimicrobial substances in food applications.

In the analysis of the literature, it clearly results that there is a strong interest in the use of cellulose and chitosan‐based active packaging. Nanocellulose, in particular, is more commonly used as filler in other biopolymers to improve dispersion of the EOs while maintaining sufficient mechanical properties. Chitosan, on the other hand, due to its intrinsic antimicrobial activity, finds applications as both support and filler.

In general, the analysis of the activity of the packaging solution is made on specific pathogens through *in vitro* tests and is often coupled with considerations about mechanical strength of the final composite. These kinds of consideration are common to about 80% of the reviewed studies, while tests on transport properties, such as water vapor and oxygen permeability of the active materials, are slightly less common.

Interestingly, about half of the studies also consider direct application to specific food for *in situ* testing of the increase in shelf life. The interest is mainly directed to meat products and fruit/vegetable products, which are the type of food that can benefit the most from this active approach due to their high perishability. Few examples of other foods, such as cheese and bakery products, were found. In all these works the deterioration of the product is monitored through laboratory analyses, and only a small percentage of them also present a sensory evaluation of the packaged food. This kind of assessment, however, is very important for the development of the final product and should be given more attention.

In the current analysis, the most commonly used EOs were, in order, oregano, cinnamon, thyme, and rosemary. However, many other plant‐derived compounds, different from EOs, were considered and were effective against a wide range of microorganisms. In fact, these natural compounds showed antimicrobial activity against the most well known gram‐positive bacteria (*S. aureus, B. subtilis, L, monocytogenes*), gram‐negative bacteria (*E. coli*), and fungi (*S. cerevisiae, A. niger*).

In general, despite the very high number of works and the potential of most of the materials considered, it is difficult to provide general guidelines for the production of active packaging based on the combination of renewable materials and essential oils. Many of the available studies are focused on very specific applications, in order to test the performance of certain essential oils, incorporated in a given materials, on the preservation of the quality of specific food. The use of a combination of essential oils in the same packaging, for example, is seldom considered, leading to ample space for further analysis and optimization of the currently tested solutions.

Most of the materials considered base their activity on the release of the active compounds but very little information is given about the release rate and kinetics, which are essential in order to design and adapt an active packaging solution for the desired shelf life. These kinds of studies, together with a more structured analysis of the interaction between the essential oils and the different matrix, would be of great interest in the selection of the best approach and the components to be used for a given application.

In conclusion, even if some applications already exist in the market, the field of active packaging has a lot of space for further research and optimization. There is a need for more structured investigations, capable of comprehending the multiple interactions existing in antimicrobial packaging, and their effects on the final properties of the film and on the final shelf life of the food.
